# Characterization of the Sweet Taste Receptor Tas1r2 from an Old World Monkey Species Rhesus Monkey and Species-Dependent Activation of the Monomeric Receptor by an Intense Sweetener Perillartine

**DOI:** 10.1371/journal.pone.0160079

**Published:** 2016-08-01

**Authors:** Chenggu Cai, Hua Jiang, Lei Li, Tianming Liu, Xuejie Song, Bo Liu

**Affiliations:** 1 Department of Bioengineering, Qilu University of Technology, Jinan, Shandong, 250353, P.R. China; 2 Department of Food Science and Engineering, Qilu University of Technology, Jinan, Shandong, 250353, P.R. China; 3 Department of Biochemistry and Molecular Biology, School of Medicine, Shandong University, Jinan, Shandong, 250012, P.R. China; Universitat Potsdam, GERMANY

## Abstract

Sweet state is a basic physiological sensation of humans and other mammals which is mediated by the broadly acting sweet taste receptor-the heterodimer of Tas1r2 (taste receptor type 1 member 2) and Tas1r3 (taste receptor type 1 member 3). Various sweeteners interact with either Tas1r2 or Tas1r3 and then activate the receptor. In this study, we cloned, expressed and functionally characterized the taste receptor Tas1r2 from a species of Old World monkeys, the rhesus monkey. Paired with the human TAS1R3, it was shown that the rhesus monkey Tas1r2 could respond to natural sugars, amino acids and their derivates. Furthermore, similar to human TAS1R2, rhesus monkey Tas1r2 could respond to artificial sweeteners and sweet-tasting proteins. However, the responses induced by rhesus monkey Tas1r2 could not be inhibited by the sweet inhibitor amiloride. Moreover, we found a species-dependent activation of the Tas1r2 monomeric receptors of human, rhesus monkey and squirrel monkey but not mouse by an intense sweetener perillartine. Molecular modeling and sequence analysis indicate that the receptor has the conserved domains and ligand-specific interactive residues, which have been identified in the characterized sweet taste receptors up to now. This is the first report of the functional characterization of sweet taste receptors from an Old World monkey species.

## Introduction

Sweet taste is a prime sense that is essential for humans and other mammals to discern and ingest sweet-tasting nutritious foods and reject environmental toxins [1]. The sweet taste perception is mediated by the broadly active class C G protein-coupled receptor (GPCR)-the sweet taste receptor heterodimer of Tas1r2 and Tas1r3. Various sweeteners including natural sugars, sweet amino acids, artificial sweeteners and sweet-tasting proteins can interact with the receptor thus induce the receptor activation and downstream signal transduction [2].

Previous studies have shown the distinct contributions of TAS1R2 and TAS1R3 subunits for the sensitivity towards various sweeteners. For example, the artificial sweeteners aspartame and neotame act on the Venus Flytrap Module (VFTM) of human TAS1R2, whereas cyclamate, neohesperidin dihydrochalcone (NHDC) and the sweet taste inhibitor lactisole interact with the transmembrane domain (TMD) of human TAS1R3 [3, 4]. The cysteine rich domain (CRD) of TAS1R3, which links the VFTM and TMD and transmits the ligand binding signal to the TMD, is proposed to mediate activation of the receptor by sweet-tasting proteins [5, 6]. Identification of the interactive site of various sweeteners in the receptor is meaningful for designing novel artificial sweet compounds.

Sweet taste preference is a species-dependent physiological process. For example, many behavioral and physiological studies have shown that the natural sugars, some amino acids, artificial sweeteners aspartame, neotame and cyclamate and sweet-tasting proteins can be perceived by catarrhines (humans, apes and Old World monkeys), but not by plathyrrhines (New World monkeys) and rodents [3–7]. Understanding the functions of sweet taste receptors from different species is essential for elucidation of the molecular mechanism of sweet taste evolution. However, only the sweet taste receptors of humans, squirrel monkey, giant panda, mice and rats have been functionally characterized until now [3–8]. The rhesus monkey (*Macaca mulatta*), which belongs to the genus *Macaca* of Old World monkeys, has been reported to respond to sweet stimuli based on the behavioral and physiological investigations [9]. However, there is no experimental data to support these findings at the molecular level until now. In this study, we cloned and functionally characterized the first sweet taste receptor Tas1r2 from an Old World monkey species, rhesus monkey.

## Materials and Methods

### Materials

HEK293E cell was purchased from Invitrogen. Aspartame, saccharin, cyclamate, sucrose, D-tryptophan, NHDC, perillartine, amiloride, monellin and thaumatin were obtained from Sigma-Aldrich. Neotame was obtained from American Health Foods & Ingredients. Stevioside was purchased from Nusci Institute & Corp. Unless stated, all other chemicals and reagents were from Sangon Biotech (Shanghai, China).

### Constructs

To obtain the rhesus monkey Tas1r2 (rhTas1r2) construct, the full coding nucleotide acid sequence of rhTas1r2 was retrieved from GenBank (DQ386298) and synthesized from the Taihe Biotechnology Co., LTD (Beijing, China) [10]. The full coding nucleotide acid sequence of rhesus monkey Tas1r3 (rhTas1r3) was not available while the experiment started. Two primers [sense (5′-CGGAATTCATGCGGCCCAGGGCAACGACCATCTGC-3′) and antisense (5′-TATTGCGGCCGCCTAGTCCCTCCTCATGGTGTAGC-3′), underlined nucleotides indicated the *EcoR*I and *Not*I restriction enzyme sites respectively)] were used to amplify the gene by PCR. The PCR product was double digested by *EcoR*I and *Not*I and then cloned into the vector pcDNA3.1. The human, squirrel monkey and mouse Tas1r2/Tas1r3 receptors (hTAS1R2/TAS1R3, smTas1r2/Tas1r3 and mTas1r2/Tas1r3) and Gα16-gust44 constructs were as described previously [6, 7, 11].

### Cell-based calcium mobilization assay

HEK293E cells were grown at 37°C in Opti-MEM (Invitrogen) supplemented with 5% fetal bovine serum (FBS). Cells were seeded onto 96-well plates and transfected with Tas1r2/Tas1r3 and Gα16-gust44 constructs using Lipofectamine 2000. The medium was replaced once after 24 h, and after an additional 24 h, the cells were washed with Hank's Buffered Salt Solution supplemented with 20 mM HEPES (HBSSH), loaded with 50 μl of 3 μM Fluo-4AM (Molecular Probe), incubated for 1 h, and then washed three times with HBSSH. Calcium mobilization was obtained with fluorescence change (excitation, 488 nm; emission, 525 nm; cutoff, 515 nm) on a FlexStation 3 system (Molecular Devices) after stimulation by 2 × tastants [12]. Each experiment was done in triplicate and the results were statistically analyzed.

### Data analysis

Data are presented as means ± S.E. of the ΔF/F value from three independent experiments. GraphPad Prism 5 software (GraphPad Software Inc.) was used for generation of bar graphs and curve-fitting (equation (log(agonist) vs. response): Y = Bottom + (Top-Bottom)/(1+10^((LogEC_50_-X))), where Top and Bottom are plateaus in the units of the Y axis, and EC_50_ is the concentration of agonist that gives a response half way between Bottom and Top. Statistical significance was determined by a two-sided unpaired *t* test.

### Molecular modeling

The sweet taste receptor Tas1r2 of rhesus monkey showed 25% and 26% overall amino acids sequence identity with the rat metabotropic glutamate receptor 1 and 3 (mGluR_1_ and mGluR_3_), respectively. We used the extracellular VFTM and CRD structures of mGluR_3_ (PDB: 2E4U) and the TMD structure of mGluR_1_ (PDB: 4OR2) as templates and link the modeled structures to simulate the overall configuration of the full length rhTas1r2 with the MODELLER9.9 program [13]. The models were evaluated by the Verify 3D server [14]. After further manual modifications (merging of fragments with best scoring models and minimizing energy by using SYBYL graphic software package (Tripos Inc.)), the model with the best Verify 3D score was selected. The sequence alignment of rhTas1r2 and the functionally characterized Tas1r2s was generated by the ClustalW program.

## Results and Discussion

### The rhesus monkey Tas1r2 responds to natural sugars and amino acids and their derivates

**Fig 1:**
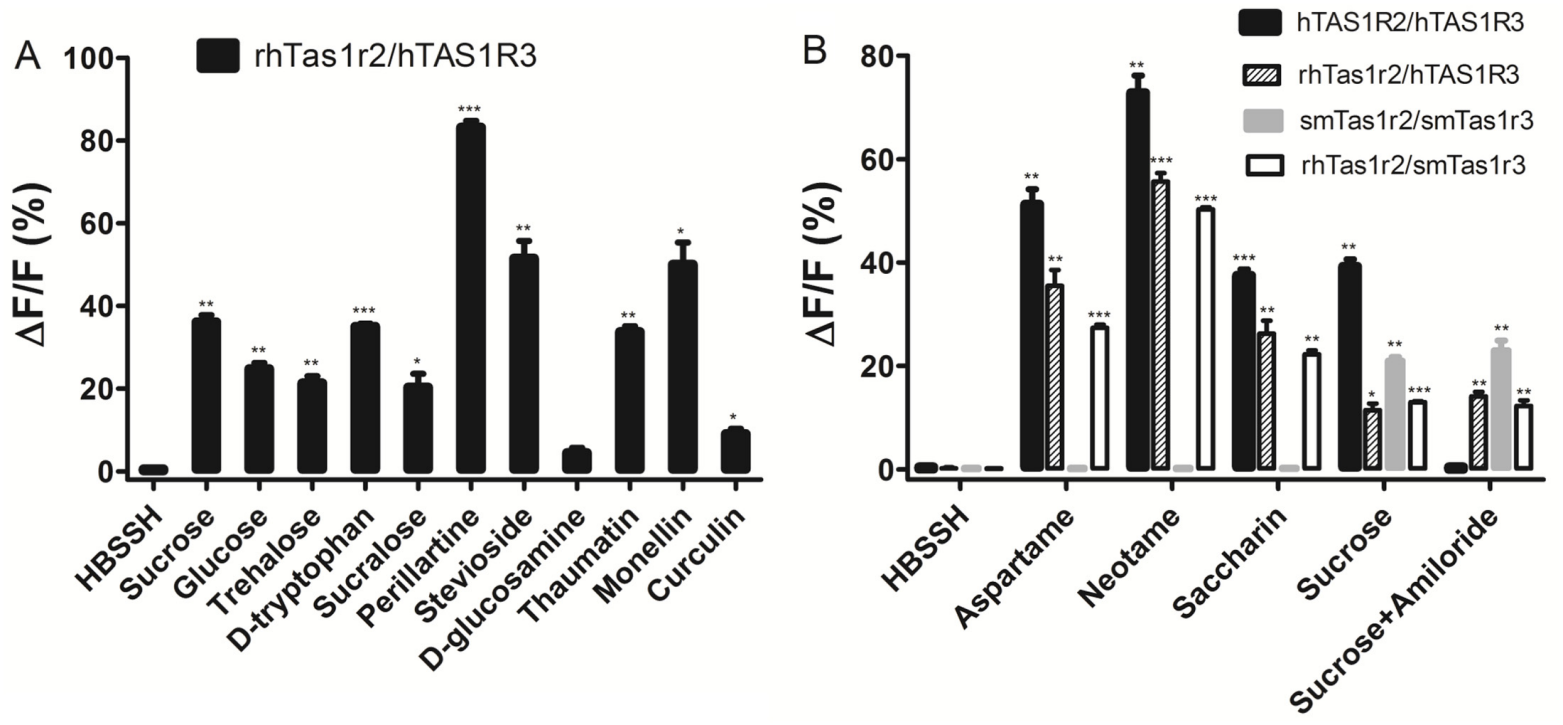
Rhesus monkey Tas1r2 responds to natural sugars, amino acids, artificial sweeteners and sweet-tasting proteins but not sweet taste inhibitor amiloride. (A) The responses of rhTas1r2/hTAS1R3 towards HBSSH (buffer solution), sucrose (300 mM), glucose (300 mM), trehalose (250 mM), D-tryptophan (5 mM), sucralose (1 mM), perillartine (300 μM), stevioside (1 mM), D-glucosamine (500 mM), monellin (45 μM), thaumatin (4.4 μM) and curculin (10 μM). (B) The responses towards HBSSH, aspartame (5 mM), neotame (0.25 mM), saccharin (1 mM) and amiloride (3 mM). The asterisks indicate significant levels of the receptor responses to various sweeteners determined by the two-sided unpaired *t* test (*: p<0.05; **: p<0.01; ***: p<0.001) compared with the receptor responses to HBSSH buffer (S1 and S2 Appendices).

The natural sugars and amino acids are two kinds of basic and widely used sweeteners in nature. Previous studies have shown that the TAS1R2 subunit is involved in the sensitivity to these sweeteners [15]. It was shown that rhTas1r2/hTAS1R3 could respond to natural sugars sucrose, glucose and trehalose, and the representative sweet amino acid D-tryptophan. The untransfected cells showed no response to any sweeteners. Moreover, rhTas1r2 could strongly respond to the derivates of sugars sucralose (substituting three of the hydroxyl groups of sucrose by chlorine atoms), perillartine (oxime of perillaldehyde) and stevioside (a glycoside of steviol), but couldn’t respond to the aminated glucose D-glucosamine (high concentration 500 mM, as shown by the statistical significance analysis in Fig 1A).

### The rhesus monkey Tas1r2 responds to the artificial sweeteners aspartame, neotame and saccharin

The dipeptide aspartame and neotame are artificial sweeteners which act on the TAS1R2 subunit of human but not New World monkeys and rodents [12]. In accordance to these findings, rhTas1r2 could strongly respond to aspartame and neotame when paired with hTAS1R3 and smTas1r3, respectively (Fig 1B).

We have demonstrated that the TAS1R2 subunit is responsible for the response towards saccharin [7]. The rhTas1r2/hTAS1R3 and rhTas1r2/smTas1r3 could respond to saccharin, while smTas1r2/smTas1r3 could not, indicating that similar to aspartame and neotame, saccharin can be perceived by Old World monkey but not by New World monkey species. These results also confirm that the Tas1r2 subunit is the molecular determinant for the sensitivity to saccharin, as replacement of the Tas1r2 subunit of squirrel monkey with that of rhesus monkey (rhTas1r2/smTas1r3) led to a gain of response to saccharin (Fig 1B).

### The rhesus monkey Tas1r2 responds to sweet-tasting proteins

It has been reported that the intensively sweet-tasting proteins can be perceived by humans, apes and Old World monkeys but not by New World monkeys and rodents [6, 10]. We described that either TAS1R2 or TAS1R3 is responsible for the sensitivity towards sweet-tasting protein monellin [7]. rhTas1r2/hTAS1R3 showed obvious sensitivity to the sweet-tasting proteins monellin and thaumatin (Fig 1A). Moreover, the sweet taste-modifying protein curculin which also elicits a sweet taste could weakly activate the rhTas1r2 receptor. These results indicated that besides humans, Old World monkey species like rhesus monkey can also respond to sweet-tasting proteins.

### Sensitivity to the sweet taste inhibitor amiloride

Amiloride is an effective inhibitor of human sweet taste receptor [16]. Our results indicated that replacement of the human TAS1R2 with the rhesus monkey Tas1r2 (rhTas1r2/hTAS1R3) resulted in a loss of the inhibition of the response to sucrose by amiloride, suggesting that the human TAS1R2 subunit is required for the sensitivity towards amiloride (Fig 1B). The functional rhesus monkey Tas1r2 receptor thus could be a useful tool to further probe the critical regions or residues determining the amiloride sensitivity located in TAS1R2.

### Species-dependent activation of the Tas1r2 receptor by an intense sweetener perillartine

**Fig 2:**
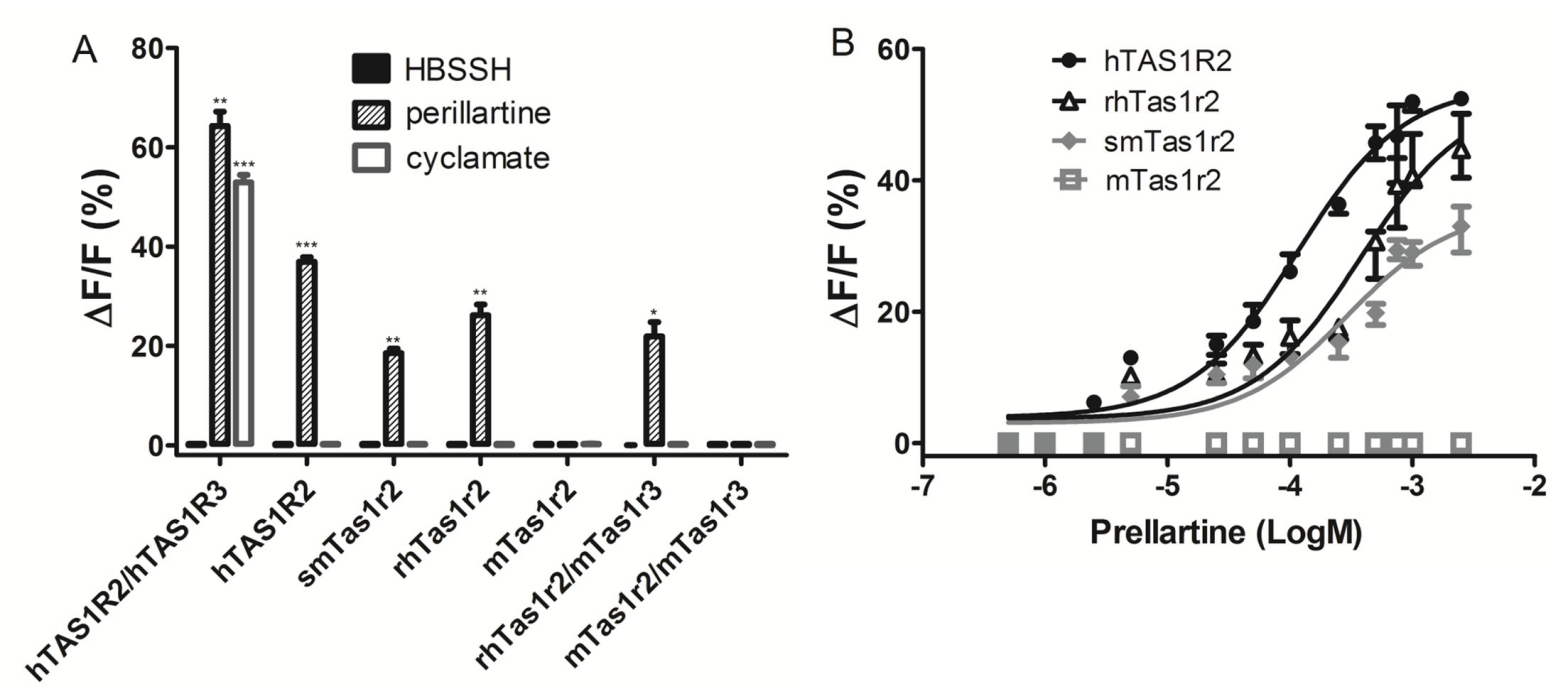
Perillartine activates the Tas1r2 subunit in a species-dependent manner. (A) Responses of the Tas1r2 subunits of human, rhesus monkey, squirrel monkey and mouse, rhTas1r2/mTas1r3 and mTas1r2/mTas1r3 towards HBSSH (buffer solution), perillartine (300 μM) and cyclamate (50 mM). The asterisks indicate significant levels of the receptor responses to the sweeteners determined by the two-sided unpaired *t* test (*: p<0.05; **: p<0.01; ***: p<0.001) compared with the receptor responses to HBSSH buffer (S3 Appendix). (B) Dose-response curve of the Tas1r2 subunits of human, rhesus monkey, squirrel monkey and mouse towards perillartine.

A sweetener perillartine has been reported to activate the human TAS1R2 (patent US 8124360). We examined the responses of the monomeric Tas1r2 subunits of human, rhesus monkey, squirrel monkey and mouse to perillartine, respectively. The human, rhesus monkey and squirrel monkey Tas1r2 subunits could be activated by perillartine, while mouse Tas1r2 could not (Fig 2A). The insensitivity of human, rhesus monkey, squirrel monkey and mouse Tas1r2 subunits to cyclamate precluded the probable involvement of Tas1r3 subunit in the assay [3]. Replacement of the mouse Tas1r2 with rhesus monkey Tas1r2 (rhTas1r2/mTas1r3) led to a gain of response to perillartine (Fig 2A). The dose-response curve showed the efficacy of responses of the Tas1r2 subunits among species: hTAS1R2>rhTas1r2>smTas1r2>mTas1r2 (Fig 2B). These results demonstrate that the monomeric Tas1r2 subunit can be activated by perillartine in a species-dependent manner.

### Molecular modeling and sequence analysis

**Fig 3:**
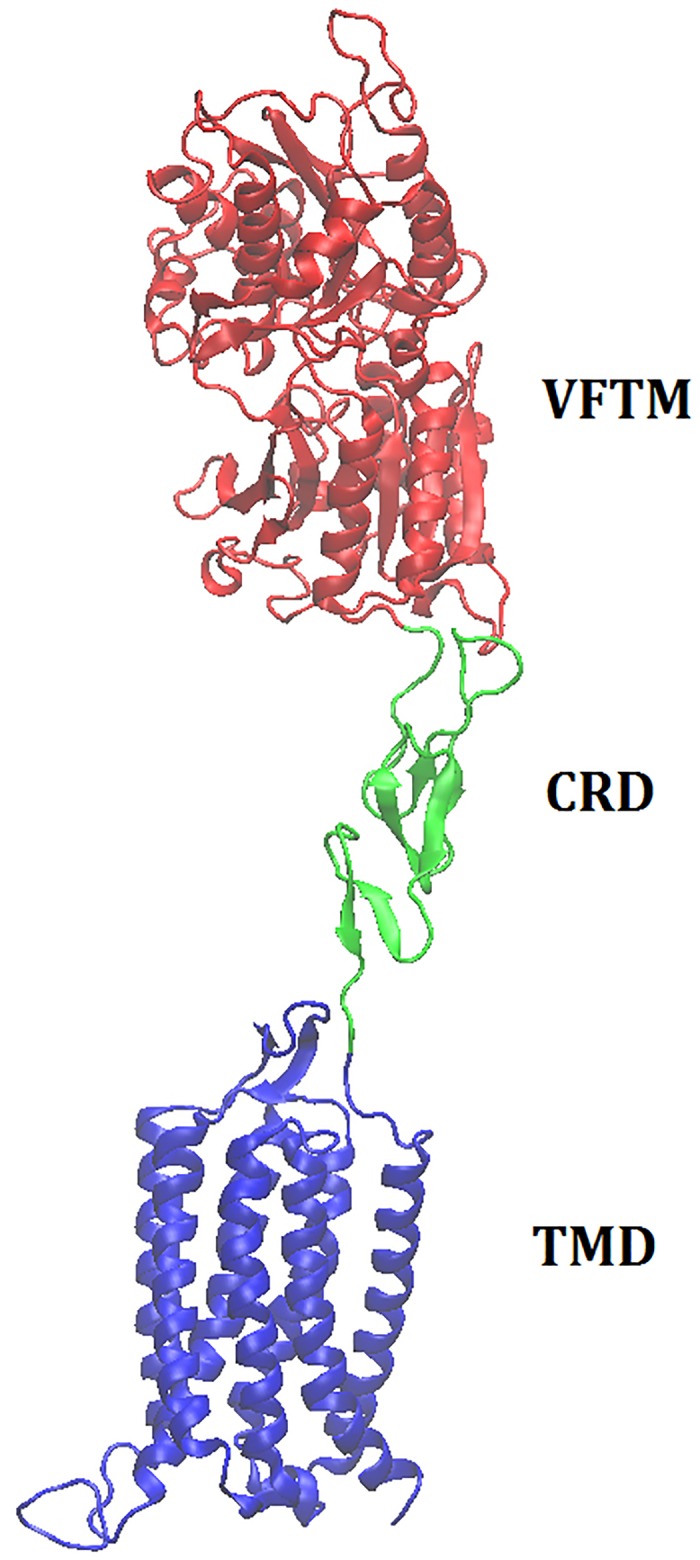
Homology modeling of the full length rhesus monkey Tas1r2. The conserved VFTM, CRD and TMD are colored in red, aqua and blue, respectively.

**Fig 4:**
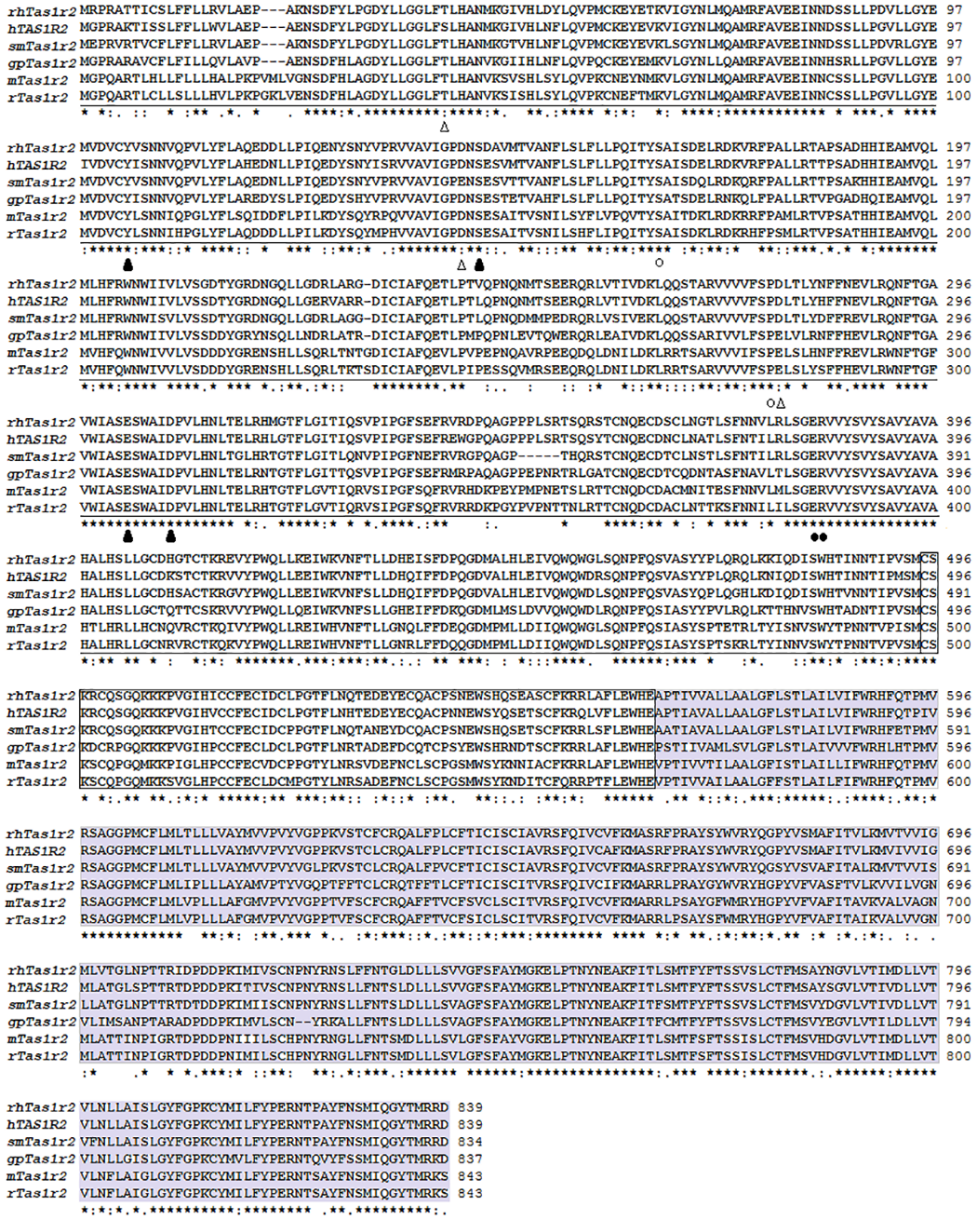
Sequence alignment of rhesus monkey Tas1r2 with the functionally characterized Tas1r2s from other species. Conserved residues are indicated by asterisk above the alignment, single and double dots represent amino acids with semi-conservative and conservative characteristics. Gaps introduced during the alignment process are indicated as dashes. The conserved VFTM, CRD and TMD regions are underlined, boxed and grey color shaded respectively. The conserved aspartame and D-tryptophan interactive residues Y103, S144, E302 and D307 (numbering based on the rhTas1r2 sequence) are denoted as black triangles, and residues T40, D142 and D278 responsible for the species-dependent taste to aspartame are denoted as white triangles. Saccharin-specific residues E382 and R383 are denoted as black cycles, and residues S165 and P277 specific for D-tryptophan or sucralose reception are denoted as white cycles [3, 12, 15]. rhTas1r2: rhesus monkey Tas1r2 (GenBank: ABD37678); hTAS1R2: human TAS1R2 (Q8TE23); smTas1r2: squirrel monkey Tas1r2 (A3QP08); gpTas1r2: giant panda Tas1r2 (XP_002926877); mTas1r2: mouse Tas1r2 (Q925I4); rTas1r2: rat Tas1r2 (Q9Z0R7).

The rhesus monkey (*Macaca mulatta*) Tas1r2 gene consists of 2520 bp coding sequence and encodes a protein of 839 amino acids (ABD37678). The protein shows 92%, 89%, 77%, 72% and 71% high overall sequence identity (EMBOSS Needle, EMBL-EBI) with the functionally characterized Tas1r2s from human (Q8TE23), squirrel monkey (A3QP08), giant panda (XP_002926877), rat (Q9Z0R7) and mouse (Q925I4), respectively. Similar to hTAS1R2 and other family C GPCRs, the modeled structure is composed of the conserved VFTM, CRD and TMD regions (Fig 3). Furthermore, sequence alignment analysis indicated that the sweeteners-specific interaction residues in hTAS1R2 are all conserved in rhTas1r2 (Fig 4). For example, residues responsible for aspartame and D-tryptophan reception E302, S144, D142, Y103, D278 and D307 (numbering according to the rhTas1r2 sequence), saccharin-specific residues E382 and R383, D-tryptophan-specific residue S165 and sucralose-specific recognition residue P277 are conserved between human TAS1R2 and rhesus monkey Tas1r2, which is consistent to the physiological responses of the two species towards these sweeteners [15]. On the other hand, residues T40, D142 and D278 are not conserved among the Tas1r2 sequences of the species studied, which is in accordance to our previous report that residue D142 is critical for the aspartame sensitivity between human and squirrel monkey, while residue T40 mediates the intensity of responses to aspartame [12]. These results imply that rhTas1r2 has the similar binding modes with those of hTAS1R2 towards various sweet ligands [3, 15]. The species-dependent response and functionally characterized receptors provide a platform to study the mechanism of evolution of sweet taste receptors.

## Conclusions

Behavioral and physiological nerve recording experiments have been performed to reveal that the sweet sense in rhesus monkey is similar to that of human but different to those of non-catarrhine primates [9]. Because most sweet ligands act on the TAS1R2 subunit of the heterodimeric receptor [15], our functional characterization of the rhesus monkey Tas1r2 at molecular level could mirror the physiological responses of rhesus monkey upon sweet stimulus mediated by Tas1r2, in spite of absence of the rhesus monkey Tas1r3. However, molecular mechanism of interaction between Tas1r2 and Tas1r3 and its potential effects on the responses towards various sweeteners cannot be demonstrated due to the absence of rhTas1r3. Nevertheless, sweet sensitivity to sweeteners targeting on Tas1r2 can be clearly evaluated with the rhesus monkey Tas1r2 [3, 4]. Furthermore, by means of the rhesus monkey Tas1r2, we revealed that residues located in Tas1r2 subunit are involved in the recognition of sweet taste inhibitor amiloride. The species-dependent activation of the monomeric Tas1r2 subunit by an intense sweetener perillartine has also been demonstrated. This first functionally characterized Tas1r2 receptor from Old World monkey species should be a useful tool not only for mapping the interactive sites in Tas1r2 towards various sweeteners, but also for further investigating the molecular mechanism of evolution of sweet taste in mammals.

## Supporting Information

S1 Appendix Statistical analysis of the rhesus monkey Tas1r2 responses towards natural sugars, amino acids and their derivates and sweet-tasting proteins. The analysis was performed with the GraphPad Prism 5 software.
(PZF)

S2 Appendix Statistical analysis of the responses towards artificial sweeteners and sweet taste inhibitor amiloride. The analysis was performed with the GraphPad Prism 5 software.
(PZF)

S3 Appendix Statistical analysis of responses of the Tas1r2 subunits of human, rhesus monkey, squirrel monkey and mouse, rhTas1r2/mTas1r3 and mTas1r2/mTas1r3 towards perillartine and cyclamate. The analysis was performed with the GraphPad Prism 5 software.
(PZF)
